# CT-Derived Sarcopenia and Outcomes after Thoracoscopic Pulmonary Resection for Non-Small Cell Lung Cancer

**DOI:** 10.3390/cancers15030790

**Published:** 2023-01-27

**Authors:** Arpad Hasenauer, Céline Forster, Johan Hungerbühler, Jean Yannis Perentes, Etienne Abdelnour-Berchtold, Joachim Koerfer, Thorsten Krueger, Fabio Becce, Michel Gonzalez

**Affiliations:** 1 Service of Thoracic Surgery, Lausanne University Hospital (CHUV), 1011 Lausanne, Switzerland; 2 Faculty of Biology and Medicine, University of Lausanne (UNIL), 1011 Lausanne, Switzerland; 3 Department of Diagnostic and Interventional Radiology, Lausanne University Hospital (CHUV), 1011 Lausanne, Switzerland

**Keywords:** non-small cell lung cancer, sarcopenia, VATS, lobectomy, segmentectomy, outcomes

## Abstract

**Simple Summary:**

Sarcopenia is defined as a progressive loss of skeletal muscle strength, mass, and function. Lung cancer patients frequently present with sarcopenia, which may be associated with poorer postoperative outcomes. This study aimed to evaluate the impact of CT-derived preoperative sarcopenia on postoperative outcomes and survival in patients that underwent thoracoscopic anatomical pulmonary resection for non-small cell lung cancer. Sarcopenia was observed in almost one-quarter of patients. Our results showed that CT-derived sarcopenia seems to have a small impact on early postoperative outcomes but no effect on overall survival. Preoperative sarcopenia screening may be a useful tool to include in the surgical risk assessment.

**Abstract:**

We aimed to evaluate whether computed tomography (CT)-derived preoperative sarcopenia measures were associated with postoperative outcomes and survival after video-assisted thoracoscopic (VATS) anatomical pulmonary resection in patients with early-stage non-small cell lung cancer (NSCLC). We retrospectively reviewed all consecutive patients that underwent VATS anatomical pulmonary resection for NSCLC between 2012 and 2019. Skeletal muscle mass was measured at L3 vertebral level on preoperative CT or PET/CT scans to identify sarcopenic patients according to established threshold values. We compared postoperative outcomes and survival of sarcopenic vs. non-sarcopenic patients. A total of 401 patients underwent VATS anatomical pulmonary resection for NSCLC. Sarcopenia was identified in 92 patients (23%). Sarcopenic patients were predominantly males (75% vs. 25%; *p* < 0.001) and had a lower BMI (21.4 vs. 26.5 kg/m^2^; *p* < 0.001). The overall postoperative complication rate was significantly higher (53.2% vs. 39.2%; *p* = 0.017) in sarcopenic patients and the length of hospital stay was prolonged (8 vs. 6 days; *p* = 0.032). Two factors were associated with postoperative morbidity in multivariate analysis: BMI and American Society of Anesthesiologists score >2. Median overall survival was comparable between groups (41 vs. 46 months; *p* = 0.240). CT-derived sarcopenia appeared to have a small impact on early postoperative clinical outcomes, but no effect on overall survival after VATS anatomical lung resection for NSCLC.

## 1. Introduction

Anatomical pulmonary resection with lymph node dissection by video-assisted thoracoscopic surgery (VATS) is recommended for early stage (I and II) and resectable locally-advanced (stage III) non-small cell lung cancers (NSCLC) [1]. The development of minimally invasive procedures has allowed a decrease in postoperative morbidity and mortality, a reduction in length of hospital stay (LOHS), and a decrease in postoperative pain [2]. Thus, surgery can now be proposed to patients that are more fragile. However, this population presents a higher risk of postoperative morbidity due to age, sex, comorbidities, and general condition [3].

Older patients with NSCLC are known to be at risk for sarcopenia, which has been identified as an independent risk factor for mortality [4,5,6,7]. Moreover, sarcopenia is associated with a higher incidence of postoperative complications after lung surgery [6,7,8,9,10]. However, all these previous studies included mainly CT measures to classify patients as sarcopenic or not, while muscle strength and function were poorly or not reported [4,6,7,8,9,10]. Sarcopenia is defined as a loss of skeletal muscle strength, mass, and function [11]; this condition is associated with infiltration of adipose and fibrotic connective tissue into muscle [12]. Several methods are used to measure skeletal muscle mass in the context of sarcopenia, including bioelectrical impedance analysis, dual-energy X-ray absorptiometry, and muscle cross-sectional area derived from computed tomography (CT) or magnetic resonance imaging (MRI). Muscle cross-sectional area at the level of the third lumbar vertebra is a validated and widely used method to assess muscle quantity and quality [11]. Muscle quantity is evaluated by the skeletal muscle area (SMA) which is normalized by patient height with the skeletal muscle index (SMI).

Because patient selection for surgery is important to reduce postoperative mortality and morbidity after VATS anatomical pulmonary resection, sarcopenia status could be a useful adjunct to identify more precisely high-risk patients. Previous studies on sarcopenia and its association with postoperative morbidity and mortality in NSCLC patients are limited by their inclusion of all stages of NSCLC and open surgical procedures [4,6,7,8,9,10].

The aim of our study was to evaluate whether CT-derived preoperative sarcopenia measures were associated with worse postoperative outcomes and survival after VATS anatomical pulmonary resection in patients with early-stage NSCLC.

## 2. Materials and Methods

### 2.1. Ethics Statement

This study was approved by the Local Ethics Committee and patient informed consent was waived (CER-VD protocol no. 2022-00741). This study is reported according to the STROBE criteria for observational studies.

### 2.2. Study Design and Patient Selection

**Figure 1:**
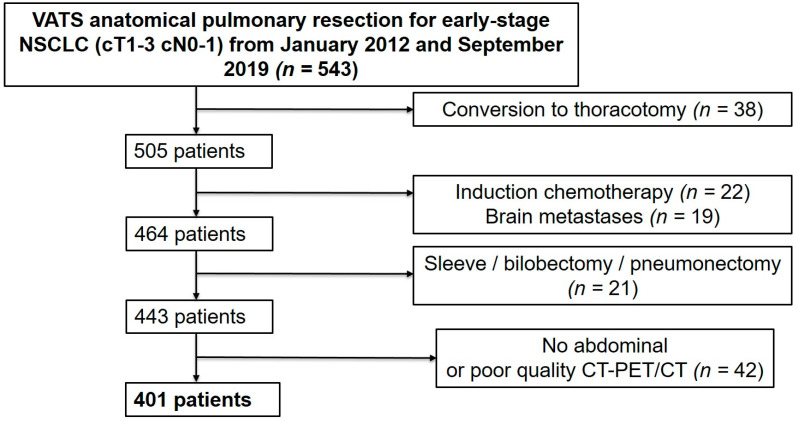
Patient selection flow chart.

This single-center retrospective study included all consecutive patients that underwent VATS anatomical pulmonary resection for early-stage and locally advanced NSCLC between 1 January 2012 and 30 September 2019 in our institution. Patients were included if they were aged ≥18 years and if they presented with NSCLC of stage I, II, or III if resectable, for which they underwent a lobectomy or segmentectomy by VATS. Patients undergoing other types of anatomical or extra-anatomical lung resection (wedge resection, bilobectomy, sleeve lobectomy, pneumonectomy), open surgical procedures, conversion thoracotomy, induction chemotherapy, those presenting with brain metastasis, and those with an absence of preoperative abdominal CT or poor-quality CT or PET/CT were excluded (Figure 1). Two groups were defined based on the presence or absence of CT-derived preoperative sarcopenia according to established cut-off values [13].

### 2.3. Data Collection

All data were retrospectively extracted from our prospective electronic database and included patient demographics, comorbidities, pulmonary functions, American Society of Anesthesiologists (ASA) score [14], Charlson comorbidity index [15,16], type of operation, tumor characteristics, and postoperative complications up to 30 days after surgery. Postoperative complications were defined as any adverse event interfering with patient management occurring during the 30-day postoperative period. The overall survival was calculated from the time of surgery to either death or last follow-up visit.

### 2.4. Surgical Procedure

There was no usual preoperative physical and mobility rehabilitation before surgery. An interdisciplinary tumor board reviewed each patient’s case to determine whether surgery was warranted. All patients underwent a preoperative cardio–respiratory physiological evaluation, which included a spirometry and a transthoracic echocardiography.

Over the entire study period, three attending specialized surgeons carried out or oversaw the various surgeries. A fourth specialized surgeon joined the staff in 2018. VATS resections were performed using general anesthesia with single-lung ventilation by double-lumen intubation. Since 2018, a uniportal approach was added to the normal anterior three-port approach. The size and location of the tumor determined the extent of the resection. For minor ground-glass opacities or peripheric solid tumors with a diameter of less than 2 cm, segmentectomy was usually performed. In the other cases, lobectomy was used to achieve anatomical pulmonary resection. Mediastinal lymph node dissection was performed in all instances.

Patients were sent to the ward after surgery. In cases of severe comorbidities or the requirement for vasoactive medications, they were transferred to the continuous or intensive care units. The interdisciplinary tumor board discussed each operated case to determine whether adjuvant therapy was necessary and to arrange subsequent follow-up.

### 2.5. Sarcopenia Measures

**Figure 2:**
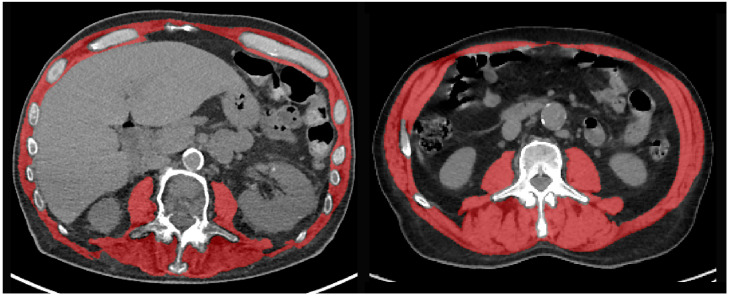
Representative examples of CT images at the L3 vertebral level with segmentation of the skeletal muscle area (red overlay) in two patients with (**left**) preoperative sarcopenia and postoperative complications, and (**right**) absence of sarcopenia and favorable outcome after VATS anatomical pulmonary resection.

Sarcopenia was quantitatively assessed on preoperative CT or PET/CT performed within the month of the planned operation. We retrospectively measured muscle mass (SMA, cm^2^) on CT or PET/CT images extracted at the mid-pedicle level of the third lumbar vertebra and including the psoas muscles, paraspinal muscles, and the muscles of the abdominal wall (Figure 2). A deep-learning-based method with a U-Net architecture algorithm was used to provide initial muscle segmentations [17,18], which has recently been validated in large datasets [19,20]. As the precision of this method cannot be guaranteed, all automated muscle segmentations were reviewed and corrected when appropriate by an attending musculoskeletal radiologist. Muscle mass was quantified using SMA and SMI. SMI was used to determine the presence/absence of sarcopenia. According to previous studies, we used the following SMI cut-off values: for women, <38.5 cm^2^/m^2^; for men, <52.4 cm^2^/m^2^ [13].

### 2.6. Statistical Analysis

Means and standard deviations (SD) were used to present continuous variables with a normal distribution. Medians and interquartile ranges (IQR) were used to describe nominal variables with several categories or continuous variables that did not follow a normal distribution. Binary variables were displayed as percentage-based figures. The unpaired Student’s *t*-test was used to test numerical variables with normal distribution, whereas the Mann–Whitney U test was used to test those without. The chi-squared test was used to test categorical variables. Statistical significance was set at a *p*-value < 0.05. Odds ratios (OR) and hazard ratios (HR) are presented with 95% confidence intervals (CI). A backward stepwise multivariate regression was performed to identify the factors associated with sarcopenia and postoperative complications. A *p*-value < 0.1 in the univariate analysis was defined as the threshold for inclusion in the multivariate analysis. Stata version 14 software (StataCorp, College Station, TX, USA) was used for statistical analyses.

## 3. Results

In total, 401 patients (female/male ratio: 173/228) with a mean age of 67.1 years (SD, 9.3 years) underwent VATS anatomical pulmonary resection for NSCLC. Sarcopenia was identified in 92 patients (22.9%). Patient characteristics are described in Table 1. Both groups were comparable in terms of age and comorbidities. Sarcopenic patients were predominantly men (75%) and had significantly lower body mass index (BMI) (mean (SD): 21.4 (3.4) kg/m^2^ vs. 26.5 (4.9) kg/m^2^; *p* < 0.001), higher ASA score (median [IQR]: 3 [2,3] vs. 2 [2,3]; *p* = 0.038), lower FEV_1_ (mean (SD): 80.7% (21.6) vs. 89.5% (21.9); *p* < 0.001), and lower DLCO (mean (SD): 63.6% (18.7) vs. 75.3% (19.7); *p* < 0.001). SMA (mean (SD): 128.1 (24) cm^2^ vs. 157.8 (36.3) cm^2^; *p* < 0.001) was significantly lower in the sarcopenic group.

Regarding postoperative outcomes (Table 2), sarcopenic patients had a longer LOD (median [IQR]: 4 [2,3,4,5,6,7] days vs. 3 [2,3,4,5] days; *p* = 0.005) and a longer LOHS (median [IQR]: 8 [5,6,7,8,9,10,11,12] days vs. 6 [4,5,6,7,8,9,10] days; *p* = 0.032). The sarcopenic group presented higher rates of overall postoperative complication (53.2% vs. 39.2%; *p* = 0.017) and pulmonary complications (48.9% vs. 33.7%; *p* = 0.008). There was no statistical difference in terms of in-hospital mortality and re-operation rate.

The univariate analysis identified several factors significantly associated with postoperative complications (Table 3): BMI < 18 kg/m^2^ (OR: 6.37, *p* < 0.001), ASA score > 2 (OR: 2.39, *p* < 0.001), sarcopenia (OR: 1.78, *p* = 0.017), Charlson comorbidity index >2 (OR: 1.52, *p* = 0.046), FEV_1_ <60% (OR: 2.4, *p* = 0.011), and DLCO < 60% (OR: 2.18, *p* = 0.001). In multivariate analysis, only two variables remained statistically significant: BMI < 18 kg/m^2^ (OR: 4.91, *p* = 0.004) and ASA score >2 (OR 1.83, *p* = 0.009).

**Figure 3:**
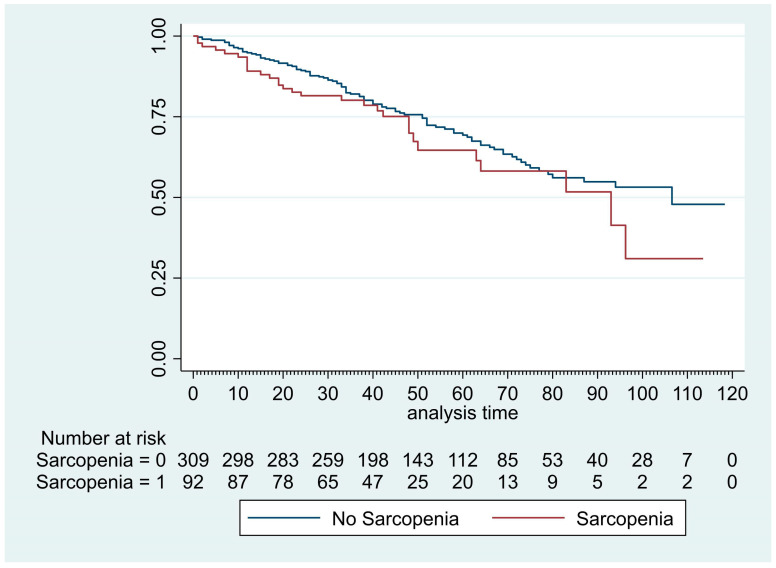
Kaplan–Meier curves of overall survival after VATS segmentectomy and lobectomy in patient with or without sarcopenia.

The median [IQR] follow-up of our patient cohort was 45 months [32.3–69]. The median [IQR] overall survival was comparable between sarcopenic and non-sarcopenic groups: 41 months [27.9–51.5] vs. 46 months [33.9–71.2], *p* = 0.24 (Figure 3). The multivariate analysis evidenced six factors associated with increased mortality (Table 4): age >70 years (HR: 1.46, *p* = 0.04), Charlson comorbidity index >2 (HR: 1.52, *p* = 0.026), pT > 1 (HR: 2.23, *p* < 0.001), pN >1 (HR: 1.79, *p* = 0.011), BMI <18 kg/m^2^ (HR: 2.01, *p* = 0.023), and DLCO < 60% (HR: 1.83, *p* = 0.002). Sarcopenia was not identified as a factor associated with increased mortality after univariate analysis (HR: 1.27, *p* = 0.240).

## 4. Discussion

In this study, we report on 401 patients who underwent VATS segmentectomy or lobectomy for early-stage NSCLC, of which almost one-quarter presented with sarcopenia. Postoperative morbidity was found to be higher in sarcopenic patients, but overall survival was similar to non-sarcopenic patients.

The prevalence of sarcopenia in the general population in the seventh decade of life is 5–13% [12], but can reach up to 50% in patients with NSCLC [10]. Prior studies have shown that in lung cancer patients, sarcopenia was an independent risk factor for shorter survival [4,5,6,7,9]. Thus, it seems important to detect sarcopenia in NSCLC patients. Although incomplete, CT or MRI assessment of body composition and muscle status at a specific level is a well-recognized and validated method to detect sarcopenia [11,21]. The advantage is that it is easily accessible and feasible as CT (and/or PET/CT) is routinely performed for NSCLC patients. However, there is no consensus on the most appropriate level to measure muscle mass. The European Working Group on Sarcopenia in Older People (EWGSOP) recommended assessing the musculature at the level of the third lumbar (L3) vertebra [11], and most studies applied this method afterwards [6,7,9,10,22,23]. Some other studies successfully assessed CT-derived sarcopenia at different vertebral levels [24]. Currently, the cut-off values used to define sarcopenia vary between studies and populations [25]. In our study, we used cut-off values previously described by other groups [13,26]. According to the EWGSOP, the current definition of sarcopenia is low muscle mass, strength, and function or low physical performance [11]. Thus, using only CT for sarcopenia assessment might misrepresent the severity of the condition. As suggested by Icard et al. [27], the definition of sarcopenia in the context of preoperative work-up of lung cancer patients should probably also include morphomic (muscle mass and BMI assessment), nutritional, and inflammation parameters. Moreover, further studies should also be performed to include patient physical capacity, strength test (grip strength and chair stand test), and performance test (gait speed, short physical performance battery, timed-up-and-go test, and 400 m walk or long-distance corridor) in sarcopenia assessment [11].

In our study, sarcopenia appeared to be more prevalent in the male population. There is currently no consensus on the male/female ratio in sarcopenia worldwide. It varies depending on the methods used to assess muscle quantity, diagnostic criteria, cut-off values, and types of population studied [28]. However, some studies have demonstrated that sexual hormones, which among others control muscle maintenance and growth, could play a role in sarcopenia etiology [29]. Hormonal changes that regulate the loss of muscle mass operate differently between men and women, which could be a reason for the difference in sarcopenia epidemiology.

Our results showed that sarcopenic patients had a lower BMI, which has also been observed by other groups [9,23,30]. An explanation for the lower BMI in sarcopenic patients might be their diagnosis of cancer, which can lead to cancer cachexia. This is characterized by involuntary loss of body weight and loss of lean body mass, due to systemic inflammation, negative protein, and energy balance [31]. However, it has been suggested that sarcopenia could also be associated with obesity and an increased fatty infiltration in the muscles [32].

We observed that sarcopenia was associated with lower pulmonary functions, which corroborates prior results by Nakada et al. [23]. As sarcopenia reduces muscle mass and strength, it can also affect respiratory muscles, a condition known as respiratory sarcopenia [33]. On the other hand, decreased pulmonary functions can also associate with sarcopenia due to limitations of mobility and general capacity leading to reduced muscle mass. This generally leads to shortness of breath, reduction of physical activity, malnutrition, and increased risk of infection. Another reason for decreased pulmonary function could be that sarcopenia is frequently associated with chronic obstructive pulmonary disease (COPD) [34,35]. This association could be due to the systemic inflammation linked to COPD [36] which favors the development of sarcopenia. All these consequences are responsible for increasing sarcopenia severity. The higher rate of pulmonary complications in sarcopenic patients could be partly explained by the difference in pulmonary functions associated with poorer pulmonary outcomes.

Several studies have shown that sarcopenia was associated with poorer postoperative outcomes after lung cancer surgery [6,7,8,9,10]. The mechanism behind this higher postoperative morbidity in sarcopenic patients is still unknown. In colorectal cancer surgery, Reisinger et al. [37] reported that sarcopenia was associated with an increased postoperative inflammatory response which could lead to a higher incidence of postoperative morbidity. Sarcopenia is a multifactorial condition and inflammatory response has effectively been described as one part of its etiology [32]. In our study, sarcopenic patients experienced increased rates of overall and pulmonary postoperative complications (mainly due to prolonged air leak), as well as longer LOD and LOHS. A higher rate of pulmonary complications in sarcopenic patients could be explained by the difference in pulmonary functions associated with poorer pulmonary outcomes. We observed an increase rate of pulmonary air leakage in sarcopenic patients which could also be explained by the decreased pulmonary functions, which is a risk factor associated with an increased rate of air leakage. However, we did not find any correlation between sarcopenia and overall postoperative complications in the multivariate analysis, as previously reported by Nakada et al. [23]. Miller et al. included 93% of VATS anatomical pulmonary resection, assessed sarcopenia via the erector spinae and pectoralis major muscles at the level of the 12th thoracic (T12) vertebra and found no significant correlation between sarcopenia and post-lobectomy morbidity or mortality [24]. Compared to studies that showed that sarcopenia independently increased the risk for postoperative complications and decreased survival in lung cancer patients [6,7,8,9,10], our study included a more strictly selected patient population undergoing a more specific surgical intervention, i.e., VATS segmentectomy or lobectomy for early-stage NSCLC. Thus, as VATS is a minimally invasive procedure known to decrease postoperative morbidity [2], this could explain the absence of association between sarcopenia and postoperative complications in our study.

We believe that future technological advances with rapid and reliable deep-learning-based automated calculation of muscle and lipid mass directly from preoperative CT data should enable wider use and inclusion of body composition measures, especially sarcopenia, in surgical risk stratification. Such a tool could allow a more precise definition of patients requiring special management or rehabilitation before surgery in order to improve their postoperative outcomes. Nevertheless, the possible correction of preoperative sarcopenia should remain individual, discussed on a case-by-case basis depending on the oncological setting.

Our study has several limitations and bias. Firstly, it is a single-center retrospective study, which inherently leads to some selection bias. Second, only SMI derived from preoperative CT data with pre-established cut-off values was used to define the presence/absence of sarcopenia. The ideal cut-off value, which would be based on our regional population, has not been determined yet. Moreover, we did not evaluate muscle strength and physical performance, which are part of the full diagnostic criteria for sarcopenia as established by the EWGSOP2 [11]. Determining sarcopenia with muscle mass alone may limit and bias the results and conclusions of our study [38]. The retrospective nature of our study did not allow the evaluation of these functional muscle parameters. However, for the same reasons, most previous studies in the field have also assessed sarcopenia using preoperative CT data alone, which makes our results more comparable to the existing literature [4,6,7,8,9,10,23,24,26,30]. Third, our median follow-up period was relatively short and is thus not representative of long-term survival. Fourth, we included different types of procedures (lobectomy and segmentectomy) and mixed lobectomy and segmentectomy procedures, as well as T4 and N2 disease which can lead to potential biases. Finally, we cannot exclude some heterogeneity in the CT and PET/CT analyses due to the different CT systems used and variability in imaging protocols over the years.

## 5. Conclusions

CT-derived preoperative sarcopenia is associated with higher rates of early overall postoperative complications, pulmonary complications, and longer LOHS and LOD; however, no association was found in multivariate analysis. Overall survival was comparable between sarcopenic and non-sarcopenic patients after VATS segmentectomy or lobectomy in our cohort of patients with early-stage NSCLC. In addition to imaging, muscle strength/function and physical performance should also be considered in future studies to assess in greater detail the preoperative surgical risk.

## Figures and Tables

**Table 1:** Patient characteristics.

Variables	All Patients (*n* = 401)	Sarcopenia (*n* = 92)	No Sarcopenia (*n* = 309)	*p*-Value
Age [years] (mean, SD)	67.1 (9.3)	67.7 (8.6)	66.9 (9.5)	0.631
Sex				
- Male	228 (43.1%)	69 (75%)	159 (51.5%)	<0.001
- Female	173 (56.9%)	23 (25%)	150 (48.5%)	
BMI [kg/m^2^] (mean, SD)	25.3 (5)	21.4 (3.4)	26.5 (4.9)	<0.001
Charlson comorbidity index (mean, SD)	2.2 (1.9)	2.1 (1.9)	2.4 (2.1)	0.177
ASA score (median, IQR)	2 (2–3)	3 (2–3)	2 (2–3)	0.038
Preoperative PFTs [%] (mean, SD)				
- FEV_1_	87.5 (21.6)	80.7 (19.3)	89.5 (21.9)	<0.001
- DLCO	72.5 (20.1)	63.6 (18.7)	75.3 (19.7)	<0.001
Comorbidities				
- Diabetes	83 (20.7%)	17 (18.5%)	66 (21.4%)	0.613
- Hypertension	221 (55.1%)	47 (51.1%)	174 (56.3%)	0.377
- Arrythmia	56 (14%)	13 (14.1%)	43 (13.9%)	0.958
SMA [cm^2^] (mean, SD)	151 (36)	128.1 (24)	157.8 (36.3)	<0.001
- Female	123.6 (20)	98.5 (9.5)	127.4 (18.3)	
- Male	171.5 (31.7)	137.4 (19.2)	186.2 (23.6)	
Type of lobectomy	304 (75.8%)	64 (69.6%)	240 (77.7%)	0.128 *
- RUL	121 (30.2%)	34 (37%)	87 (28.2%)	
- RML	25 (6.2%)	4 (4.3%)	21 (6.8%)	
- RLL	48 (12%)	9 (9.8%)	39 (12.6%)	
- LUL	69 (17.2%)	12 (13%)	57 (18.4%)	
- LLL	41 (10.2%)	5 (5.4%)	36 (11.6%)	
Segmentectomy	102 (25.4%)	29 (31.5%)	73 (23.6%)	0.133
- Simple	65 (16.2%)	18 (19.6%)	47 (15.2%)	
- Complex	37 (9.2%)	11 (12%)	26 (8.4%)	
Combined procedure (segmentectomy + lobectomy)	5 (1.2%)	1 (1.1%)	4 (1.3%)	NA
TNM staging (8th edition)				
- pT1	201 (50.1%)	45 (48.9%)	156 (59.5%)	0.738 *
- pT2	148 (36.9%)	32 (34.8%)	116 (37.5%)	
- pT3	37 (9.2%)	11 (12%)	26 (8.4%)	0.517 *
- pT4	15 (3.7%)	4 (4.4%)	11 (3.6%)	
- pN0	341 (85%)	79 (85.9%)	262 (84.8%)	
- pN1	31 (7.7%)	5 (5.4%)	26 (8.4%)	
- pN2	28 (7%)	8 (8.7%)	20 (6.5%)	
Histology Adenocarcinoma	291 (72.6%)	61 (66.3%)	230 (74.4%)	
Squamous cell carcinoma	91 (22.7%)	24 (26.1%)	67 (21.7%)	0.126 *
Others	19 (4.7%)	7 (7.6%)	12 (3.9%)	
Size [mm] (mean, SD)	25.8 (16.2)	27.3 (17.1)	25.4 (15.9)	0.317
R0	400 (99.7%)	91 (98.9%)	309 (100%)	1
R1	1 (0.3%)	1 (1.1%)	0 (0%)	

SD: standard deviation; BMI: body mass index; ASA: American Society of Anesthesiologists; IQR: interquartile range; PFTs: pulmonary function tests; FEV_1_: forced expiratory volume in one second; DLCO: diffusing capacity of the lung for carbon monoxide; SMA: skeletal muscle area; HU: Hounsfield unit; RUL: right upper lobe; RML: right middle lobe; RLL: right lower lobe; LUL: left upper lobe; LLL: left lower lobe; pTNM: primary Tumor (T), lymph Nodes (N), and Metastasis (M) staging after histopathologic examination (p); R0: complete tumor resection; R1: microscopic residual tumor; * chi-squared, NA: not applicable.

**Table 2:** Postoperative complications.

Variables	All Patients (*n* = 401)	Sarcopenia (*n* = 92)	No Sarcopenia (*n* = 309)	*p*-Value
Length of drainage [days] (median, IQR)	3 (2–6)	4 (2–7)	3 (2–5)	0.005
Length of hospital stay [days] (median, IQR)	7 (4–10)	8 (5–12)	6 (4–10)	0.032
Overall complications (30 days)	170 (42.4%)	49 (53.2%)	121 (39.2%)	0.017
Cardiac complications	30 (7.5%)	7 (7.6%)	23 (7.4%)	0.767
Pulmonary complications	149 (37.2%)	45 (48.9%)	104 (33.7%)	0.008
Pneumonia	76 (18.9%)	20 (21.7%)	56 (18.1%)	0.438
Pneumothorax	13 (3.2%)	3 (3.3%)	10 (3.2%)	0.991
Empyema	3 (0.7%)	0 (0%)	3 (1%)	1
Hemothorax	2 (0.5%)	1 (1.1%)	1 (0.3%)	1
Prolonged air leak (>5 days)	103 (25.7%)	34 (37%)	69 (22.3%)	0.005
ARDS	2 (0.5%)	1 (1.1%)	1 (0.3%)	1
Subcutaneous emphysema	25 (6.2%)	7 (7.6%)	18 (5.8%)	0.536
In-hospital postoperative mortality	1 (0.2%)	0 (0%)	1 (0.3%)	1
Re-operation	11 (2.7%)	3 (3.3%)	8 (2.6%)	0.730

IQR: interquartile range; ARDS: Acute respiratory distress syndrome.

**Table 3:** Univariate and multivariate analyses of factors associated with overall postoperative complications.

Variables	Univariate			Multivariate		
	OR	95% CI	*p*-Value	OR	95% CI	*p*-Value
Age > 70 years	1.01	0.99–1.03	0.224			
Sex (female)	0.67	0.45–1.01	0.057	0.86	0.54–1.36	0.515
BMI < 18 kg/m^2^	6.37	0.91–0.98	<0.001	4.91	1.67–14.43	0.004
ASA > 2	2.39	1.57–3.47	<0.001	1.83	1.16–2.91	0.009
SMA	0.99	0.99–1.00	0.331			
Sarcopenia	1.78	1.11–2.82	0.017	1.12	0.65–1.92	0.689
Charlson comorbidity index > 2	1.52	1.01–1.24	0.046	1.18	0.75–1.84	0.469
Segmentectomy	1.09	0.69–1.72	0.683			
FEV_1_ < 60%	2.40	1.22–4.74	0.011	1.33	0.63–2.83	0.454
DLCO < 60%	2.18	1.40–3.38	0.001	1.57	0.96–2.56	0.068

OR: odds ratio; CI: confidence interval; BMI: body mass index; ASA: American Society of Anesthesiologists; FEV_1_: forced expiratory volume in one second; DLCO: diffusing capacity of the lung for carbon monoxide.

**Table 4:** Univariate and multivariate analyses of factors associated with mortality.

Variables	Univariate			Multivariate		
	HR	95% CI	*p*-Value	HR	95% CI	*p*-Value
Age > 70 years	1.39	0.98–1.97	0.057	1.46	1.01–2.10	0.04
Sex (female)	0.71	0.49–1.02	0.064	0.80	0.54–1.18	0.268
Charlson comorbidity index > 2	1.71	1.21–2.42	0.002	1.52	1.05–2.19	0.026
Sarcopenia	1.27	0.84–1.92	0.240			
pT > 1	1.96	1.37–2.79	<0.001	2.23	1.54–3.23	<0.001
pN > 1	1.59	1.03–2.47	0.036	1.79	1.14–2.82	0.011
Segmentectomy	0.81	0.52–1.27	0.366			
BMI < 18 kg/m^2^	1.84	1.04–3.28	0.036	2.01	1.10–3.68	0.023
ASA > 2	1.95	1.36–2.76	<0.001	1.28	0.85–1.94	0.231
FEV_1_ < 60%	1.91	1.13–3.23	0.016	1.63	0.91–2.91	0.102
DLCO < 60%	2.21	1.56–3.14	<0.001	1.83	1.24–2.70	0.002

HR: hazard ratio; CI: confidence interval; pTNM: primary Tumor (T), lymph Nodes (N), and Metastasis (M) staging after histopathologic examination (p); BMI: body mass index; ASA: American Society of Anesthesiologists; FEV_1_: forced expiratory volume in one second; DLCO: diffusing capacity of the lung for carbon monoxide.

## Data Availability

The data that support the findings of this study are available from the corresponding author upon reasonable request.
